# Effects of lemon verbena extract (Recoverben®) supplementation on muscle strength and recovery after exhaustive exercise: a randomized, placebo-controlled trial

**DOI:** 10.1186/s12970-018-0208-0

**Published:** 2018-01-23

**Authors:** Sybille Buchwald-Werner, Ioanna Naka, Manfred Wilhelm, Elivra Schütz, Christiane Schoen, Claudia Reule

**Affiliations:** 1 Vital Solutions GmbH, Hausingerstrasse 6, 40764 Langenfeld, Germany; 2 Ulm University of Applied Sciences, Albert-Einstein-Allee 55, 89081 Ulm, Germany; 3 BioTeSys GmbH, Schelztorstrasse 54-56, D-73728 Esslingen, Germany

**Keywords:** Sports nutrition, Lemon verbena, *Aloysia citriodora*, Recoverben®, Muscle strength, Recovery, Exhaustive exercise, eiMD, Muscle soreness, Glutathione peroxidases

## Abstract

**Background:**

Exhaustive exercise causes muscle damage accompanied by oxidative stress and inflammation leading to muscle fatigue and muscle soreness. Lemon verbena leaves, commonly used as tea and refreshing beverage, demonstrated antioxidant and anti-inflammatory properties. The aim of this study was to investigate the effects of a proprietary lemon verbena extract (Recoverben®) on muscle strength and recovery after exhaustive exercise in comparison to a placebo product.

**Methods:**

The study was performed as a randomized, placebo-controlled, double-blind study with parallel design. Forty-four healthy males and females, which were 22–50 years old and active in sports, were randomized to 400 mg lemon verbena extract once daily or placebo. The 15 days intervention was divided into 10 days supplementation prior to the exhaustive exercise day (intensive jump-protocol), one day during the test and four days after. Muscle strength (MVC), muscle damage (CK), oxidative stress (GPx), inflammation (IL6) and volunteer-reported muscle soreness intensity were assessed pre and post exercise.

**Results:**

Participants in the lemon verbena group benefited from less muscle damage as well as faster and full recovery. Compared to placebo, lemon verbena extract receiving participants had significantly less exercise-related loss of muscle strength (*p* = 0.0311) over all timepoints, improved glutathione peroxidase activity by trend (*p* = 0.0681) and less movement induced pain (*p* = 0.0788) by trend. Creatine kinase and IL-6 didn’t show significant discrimmination between groups.

**Conclusion:**

Lemon verbena extract (Recoverben®) has been shown to be a safe and well-tolerated natural sports ingredient, by reducing muscle damage after exhaustive exercise.

**Trial registration:**

The trial was registered in the clinical trials registry (clinical trial.gov NCT02923102). Registered 28 September 2016

## Background

All kinds of training, moderate, exhaustive or unaccustomed, cause so-called exercise-induced muscle damage (eiMD) accompanied by oxidative stress and inflammation [1]. Delayed onset muscle soreness (DOMS) is the most common symptom of eiMD, whereas histological evidence of disruption of the myofibrillar structure and myofibre necrosis, as well as inflammation, are the ultimate signs of eiMD [1]. DOMS is associated with muscle fatigue and muscle soreness, symptoms, which last for a number of days and have a negative impact on the exercise performance of athletes and amateur sports people, especially when they carry out long-term training programs [1]. Incomplete regeneration of myofibrillar structures and metabolic processes before the next training increases the risk for secondary injuries. Therefore, it is important for the adherence to training plans and for the training success that regeneration is as effective and as short as possible.

A product that could accelerate recovery from DOMS or muscular fatigue would be beneficial not only for high performance athletes, but also for amateur athletes, enabling them to train more frequently or reduce the risk of injury. As inflammation and ROS (reactive oxygen species) are presumably the main cause of DOMS [1], it is hypothesized that natural ingredients with anti-inflammatory and antioxidative properties may help in accelerating or supporting the regeneration after muscle damage inducing exercise [2–4]. The use of supplements with antioxidative or anti-inflammatory effects in the sports nutrition is already widespread. Many of these ingredients and products have been investigated in recent years; for example, curcumin [5], omega-3 fatty acids [6, 7] and polyphenols [2, 8–10].

Lemon verbena (*Aloysia citriodora (L.)*) is an annual eatable herbaceous plant native to South America commonly used as tea, refreshing beverage, food, or spice. Traditional medicinal applications are related to digestion and nervous discomfort [11]. A literature search focusing on peer-reviewed publications showed that only limited data are available for lemon verbena in connection with muscular recovery after exercise. PubMed listed over 80 publications in June 2017, that investigated lemon verbena as stand-alone product or in combination with other ingredients in any context (keywords for search were *Aloysia citriodora*, *Lippia citriodora*, *Lippia triphylla*, *Aloysia triphylla,* and lemon verbena).

Analytical references demonstrate that water-based extracts out of lemon verbena leaves are high in polyphenols [12, 13]. Several in vitro and in vivo studies have shown antioxidative and anti-inflammatory effects [11, 14–18]. Out of six human studies [19–24], only one human pilot study was published investigating lemon verbena extract on the muscular damage biomarker, creatine kinase and liver biomarker related to oxidative stress [19]. This study showed some effects on cytokines and oxidative stress markers in neutrophils, but no functional parameters like muscle strength or muscle soreness were essessed. Furthermore, this study used a high dosage of 1800 mg/day which is not suitable for application in food supplements as it would require the intake of approximatley nine capsules per day. Therefore, further research to observe effects of lemon verbena on muscle damage, muscle soreness, and recovery needs to be performed.

A proprietary lemon verbena extract (Recoverben®), high in polyphenols, was developed and recently identified as an anti-inflammatory agent [25]. One mode of action responsible for the anti-inflammatory properties is the inhibition of cyclooxygenase (COX) [26]. COX inhibition properties have also been shown for curcumin [27], which is also an natural ingredient shown to be beneficial in recovery [5, 28]. Based on these data and on data from literature, we hypothesized that a lemon verbena extract (Recoverben®) could have beneficial effects on exercise induced muscle damage, muscle soreness, and recovery. The aim of this study was to investigate effects on functional, metabolic, and subjective parameters of recovery. Furthermore, parameters indicating antioxidative and anti-inflammatory properties were included to document mode of action.

## Methods

### Study design

This study was a double blind, randomized controlled trial with a parallel-group design that investigated the effects of a proprietary lemon verbena extract (Recoverben®) supplementation on muscle strength and recovery after exhausive exercise. It was conducted in orientation to the ICH-GCP guidelines, in compliance with the declaration of Helsinki, and was reviewed by the Institutional Review Board (IRB) “Landesärztekammer Baden Württemberg” without concerns (F-2016-080 September 13th, 2016). All subjects signed the IRB-approved informed consent prior to any procedures. The study was performed from October 2016 to March 2017 at BioTeSys GmbH, Esslingen, Germany, an independent study site which is focused on nutritional research.

### Subjects

Subjects were recruited from internal database of the study site, advertisements in local newspapers, and notice boards in public buildings. Seventy subjects responded to the advertising campaign and received detailed information about the study. Out of these, 45 subjects were invited for screening visits. Forty-four healthy, non-smoking, moderately active (exercise 1–3 times per week) men and women with an age between 22 and 50 years and a BMI between 19 and 30 kg/m^2^ were deemed eligible for the current study. Subjects had a usual intake of five or less portions of fruits plus vegetables per day. Detailed inclusion- and exclusion criteria are presented in Table 1. Eligibility was evaluated by medical history, concomitant medication, physical examination, electrocardiogram, blood pressure, and anamnesis. Physical activity was assessed using the International Physical Activity Questionnaire (IPAQ) [29]. Nutrition pattern of subjects was determined during the screening visit using a subjective, retrospective, semi-quantitative nutrition frequency questionnaire. The score ranges from 0 to 100, whereby 0 means a very good nutrition pattern and 100 a very poor nutrition pattern [30]. The questionnaire was developed for the German-speaking area and tested in a neutral and geriatric collective. Adequate determination of supply of micronutrient was validated by comparing with a detailed quantitative food frequency questionnaire [30]. A score around 50 can be interpreted as balanced nutrition. Amount of fruits and vegetables portions taken per day was asked during screening, whereby one portion was specified to be around 150 g. Subjects were requested to refrain from intake of anti-inflammatory or antioxidative drugs or supplements during the study, potentially interfering with this trial. They were asked not to change their dietary habits and physical activity during the study. The evening before the study days, subjects ate a standardized dinner low in polyphenols (noodles with cheese sauce). During the study days, they received a standardized breakfast (cereal bars) and a standardized snack (white wheat roll with butter and cheese). Amount and time were also standardized. Alcohol intake and exhaustive activities were prohibited 48 h before the study days until 96 h after the exhaustive exercise. Subjects were asked for changes in nutrition habits and sportive activity at the end of the study.

**Table 1:** In- and exclusion criteria

Inclusion criteria	Exclusion criteria
Subject is able and willing to sign the Informed Consent Form prior to screening evaluations	Relevant history, presence of any medical disorder or chronic intake of medication/dietary supplements (e.g. polyphenols, anti-inflammatory or antioxidative drugs or supplements, antihypertensive drugs) potentially interfering with this study at screening
Healthy subjects: Subject is in good physical and mental health as established by medical history, physical examination, electrocardiogram, vital signs, results of biochemistry and haematology	For this study clinically relevant abnormal laboratory, vital signs or physical findings at screening
Men and women	Diabetes or serious cardiovascular diseases
Age ≥ 22 and ≤50 years	Change of dietary habits within the 2 weeks prior to screening (for instance start of a diet high in vegetables and fruits (≥ 5 portions per day)
BMI: 19–30 kg/m^2^	Diet high in vegetables and fruits ≥ 5 portions per day
Physically active 1–3 times per week	Participants anticipating a change in their lifestyle or physical activity levels during the study
Nonsmoker	Subjects not willing to abstain from intake of analgesic medication (e.g. Aspirin) 24 h prior to visit 2 until visit 5
Able and willing to follow the study protocol procedures	Subjects with history of drug, alcohol or other substances abuse, or other factors limiting their ability to co-operate during the study
	Known hypersensitivity to the study preparation or to single ingredients
	Pregnant subject or subject planning to become pregnant during the study; breast-feeding subject
	Known HIV-infection
	Known acute or chronic hepatitis B and C infection
	Blood donation within 4 weeks prior to visit 1 or during the study
	Subject involved in any clinical or food study within the preceding month

**Fig. 1:**
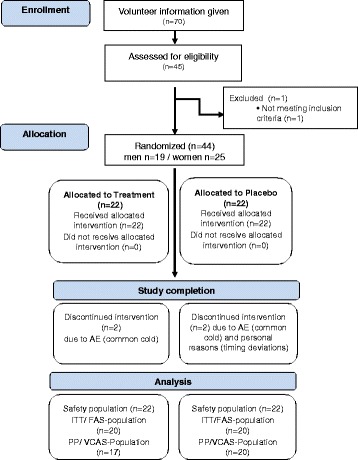
Dispostion of subjects following Concort

Subjects were randomly assigned to the study groups and stratified by gender after assessing eligibility during screening. To ensure double-blind performance, the randomization scheme was created by the sponsor using the software Randlist. All subjects, the investigator, and study staff involved in study performance, and data analysis were blinded until database lock. Disposition of subjects is summarized in Fig. 1.

### Study product and supplementation

The investigated commercial product Recoverben® (batch number 16P0007) is a proprietary lemon verbena extract obtained by water extraction out of organic dried lemon verbena leaves. Lemon verbena (*Aloysia citriodora (L.)*) is a member of the family Verbenaceae. The product is a native extract without any additives, standardized to more than 18% polyphenols. The extract is hydrophilic and can easily be dissolved in water, and has a high ORAC level of 170.000 μmol TE/100 g. Lemon verbena extract (Recoverben®) as well as the placebo were formulated in capsules, matching in size and color, and were supplied by Vital Solutions GmbH, Germany. Each capsule contained 200 mg lemon verbena extract or placebo (maltodextrin). All subjects were instructed to take two capsules daily in the morning. Products were consumed 10 days before an exhaustive exercise test, during the test day and four days after the test. The ingredient is safe for human consumption and its quality complies with EU legislation concerning hygiene, contaminants, and maximum residue levels of pesticides of foodstuff [31, 32]. Additionally, the extract has been tested for and shown to be free of banned substances by a specialist anti-doping laboratory (LGC Limited, UK), therefore demonstrating suitability as a sports nutrition product.

### Exhaustive exercise protocol

In the current study, maximal eccentric loading of the lower extremity was induced by an intensive jump-protocol 10 days after the start of supplementation, which was modified based on a jump protocol used previously [33]. The protocol comprised 200 countermovement jumps with an additional load of 10% of the participant’s body weight. The 200 countermovement jumps were performed in 10 sets of 20 jumps every four seconds with 90 s rest between sets. Knee joint angle between the jumps had to be 90°, which was controlled by the observer. At the end of the test, rating of perceived exertion (RPE) was obtained using the Borg RPE scale [34]. Borg RPE scale and jump repetitions were used to ensure subjective exhaustion after exercise and to control comparability of jump protocol between groups. Massive deviation from jump protocol was defined as exclusion criteria from VCAS analysis for exclusion of confounding factors.

### Muscle function testing - maximal voluntary contraction (MVC)

Changes in muscle function appear to be the best marker for the degree of exercise induced muscle damage [1]. Therefore, in the current study, MVC was investigated by assessing isometric strength of the *M. quadriceps* femoris, with 90° knee angle, using a dynamometer KM40 (2kN) from ME-Messsysteme GmbH and a strength chair from fasttwitch, TTI GmbH. Subjects were fixed in a seated position with a hip belt, had their arms crossed in front of the chest, and had the free leg hanging without contact to any surface to reduce support by other body parts during the test. Before each measurement, subjects performed a 3 min warm-up (level 7, 70 rpm) on a cycle ergometer (Crane Power Studio Ergometer). The strength measurement was performed for the dominant leg. This was identified at screening by a shove-test, where the subject was pushed unexpected by the observer and the leg which was used by subject to balance was defined as dominant leg. All examinations were performed in triplicates, immediately before and 3, 24, and 48 h after the exhaustive exercise protocol. The highest value was used for analysis. Subjects were familiarized to the measurement at screening. For each subject, the settings of the strength chair (position of back rest, leg rest and position of measurement arm) were documented. All further measurements were performed with the individual settings. First measurement was performed during screening visit to avoid training effects during study visits. Variability was checked between screening and pre-exercise measurement.

### Perceived muscle soreness

Muscle soreness was measured using two different methods.

#### Movement induced pain (VAS)

Subjects were asked to sit down into and get up from a chair and to rate the pain they experienced in doing so using a 100 mm visual analogue scale (VAS), which consisted of a from zero mm (no pain) to 100 mm (worst imaginable pain). This assessment was conducted immediately before and 3, 24, 48, 72, and 96 h after the exhaustive exercise protocol. Using VAS is frequently described in literature for assessing acute exercise induced pain [2, 33, 35].

#### Retrospective pain (Likert scale)

A seven point retrospective pain questionnaire (7 point Likert-scale for muscle soreness) by Vickers et al. was used to evaluate retrospective perceived pain during daily life activities with zero “a complete absence of pain” and six “a severe pain that limits my ability to move” [36]. The subjects were asked to answer the questionnaire immediately before the exhaustive exercise and 24, 48, 72, and 96 h after the jump test.

### Biochemical analysis

Different biomarkers were analyzed to evaluate muscle damage and antioxidative capacity to describe exercise-induced oxidative stress.

#### Creatine kinase (CK)

CK is a biomarker for muscle damage typically increased after intense exervcise. In our study, CK was determined from blood samples obtained before and 3, 24, and 48 h after the exhausting exercise protocol. Analyses were carried out at Synlab Medizinisches Versorgungszentrum Leinfelden using an enzymatic-kinetic test method [37].

#### Glutathione peroxidase (GPx)

Exercise training is accompanied with oxidative stress via production of reactive oxygen species (ROS), and modulating the endogenous antioxidant defense system, including GPx. In a healthy organism, exercise induces GPx levels, inactivating ROS and maintain them in physiological levels [38, 39]. The determination in plasma (GPxP) was carried out at the study lab via GPx-Assay-Kit (Cayman Chemical Company, Ann Arbor, MI, USA) pre-exercise and 3, 24, and 48 h post exercise.

#### Interleukin-6 (IL-6)

IL-6 is a multifunctional cytokine involved in pro- as well as anti-inflammatory processes. Exercise-induced IL6 response is dependent on intensity and duration of the exercise [40]. The determination in serum was carried out at the study lab via Quantikine® HS Human IL-6 Immunoassay Kit (R&D Systems, Inc., MN, USA) pre-exercise and 3, 24, and 48 h post exercise.

### Safety and tolerability

At each visit, changes in physical conditions since the last visit were reviewed with subjects. Based on entries in subject diaries, complete blood count, and adverse events were assessed. Tolerability of the study product was assessed 96 h after exercise, and at the end of study. The subjects rated overall tolerability by selecting out of three categories: “well tolerated”, “slightly unpleasant”, and “very unpleasant”.

### Statistics

The study was planned as an exploratory trial. Sample size was calculated based on different studies observing the effects of lemon verbena and using a similar design [19, 35]. Therefore, a sample size calculation was performed with effect size f = 0.2, significance level 0.05, power 80%, two number of groups and four measurement time points (pre, 2 h, 24 h, 48 h), correlation among repeated measures 0.5 and with nonsphericity correction 1. With these suggestions, a total sample size of 36 subjects was calculated. Considering a drop-out rate of 10%, 40 subjects were planned to be included in the study.

Objectives were the difference of muscle strength, muscle soreness, retrospective pain, CK, IL-6, and glutathione peroxidase after exhaustive exercise under lemon verbena in comparison to placebo.

Data were analyzed using SAS Version 9.3 and GraphPad Prism Version 5.04. All statistical tests were performed two-sided. Significance level was set to 0.05. For evaluation of treatment effects, a linear mixed model with repeated measures was used. For retrospective pain, a generalized linear mixed model with Poisson-distribution for count data was used. Due to explorative data analysis, no correction for multiple comparison was performed. Gender, its interaction with treatment and the respective pre value was included as covariates for the biomarkers and MVC. For movement induced pain and retrospective pain only gender and its interaction with treatment were defined as covariates. Changes within groups were assessed using one way ANOVA or Friedmann test, as appropriate. All efficacy parameters were checked for baseline differences. Results presented below refer on valid case analysis set (VCAS). VCAS criteria were pre-defined in the protocol: missing data, adverse events or concomitant medication interfering with study results, extreme outliers, and major protocol violation (compliance <85%, > 115%, drop outs / withdrawals, major deviation of study performance).

## Results

### Group characteristics

Out of 44 subjects allocated to intervention, 40 subjects (19 men and 21 women) completed the study according to the protocol (full analysis set; FAS). Four subjects dropped out after start of supplementation and before efficacy testing. Three of these suffered from a common cold, which did not allow them to perform the exhaustive exercise test, and one dropped out due to personal decision.

Thirty-seven subjects were analyzed for valid case analysis set (VCAS). One subject had a strong common cold during the exhaustive exercise test, which may have influenced biomarker results. Another subject did not meet compliance criteria for study product intake and the third subject had major deviation of the exhaustive exercise protocol. As these three subjects did meet exclusion criteria for VCAS analysis, they were not considered in final analysis (VCAS).

Gender, age, BMI, blood routine markers, and blood pressure did not differ significantly between the groups prior to the study. Subject characteristics are summarized in Table 2. The nutrition frequency questionnaire scores were 45.35 ± 10.51 points in the lemon verbena group and 42.65 ± 11.33 points in the placebo group (*p* = 0.4597). Most subjects consumed between two and four portions fruits plus vegetables per day (lemon verbena: 82%, placebo: 70%). Intake of fruit and vegetables, as supplier for polyphenols, were comparable between groups. The protocol compliance of study product intake was very good. For VCAS, all subjects met the compliance criterion ≥85% and ≤115% of study preparation consumed (lemon verbena group: 102 ± 5%, placebo: 101 ± 2%). For FAS, one subject had a compliance >115% since much less products remained, bringing up uncertainty about correct intake, which was the reason for exclusion from VCAS. Borg scale data after exhaustive exercise and jump repetitions were not significantly different between groups (*p* = 0.8997, *p* = 0.1561). Therefore, burden of subjects by the exhaustive exercise and jump protocol was comparable between groups, so a comparable stress could be expected.

**Table 2:** Subject characteristics at screening for FAS (*N* = 40, 19 men, 21 women)

Parameter [unit]		Lemon verbena (*N* = 20 59% men, 41% women)	Placebo (*N* = 20 40% men, 60% women)	Inclusion criteria/Reference range
Age [years]	Mean	31.7	30.6	22–50
	Sd	8.8	9.3	
BMI [kg/m^2^]	Mean	22.67	23.0	19–30
	Sd	2.3	2.5	
Activity [MET min/week]	Mean	3209	2470.0	–
	Sd	3957.0	2521	
CHOL [mg/dL]	Mean	172.5	180.0	100–200
	Sd	23.0	31.85	
TG [mg/dL]	Mean	89.68	82.14	<160
	Sd	43.53	24.71	
HDL [mg/dL]	Mean	58.91	58.95	>40
	Sd	6.487	14.24	
LDL [mg/dL]	Mean	93.86	109.5	<150
	Sd	16.32	28.7	
Glucose [mg/dL]	Mean	87.55	86.14	60–100
	Sd	8.405	4.96	
SBP [mmhg]	Mean	121.6	121.6	120–129
	Sd	12.8	12.8	
DBP [mmhg]	Mean	75.8	70.6	80–84
	Sd	8.8	10.5	

*BMI* body mass index, *MET* metabolic equivalent of task, *CHOL* cholesterol, *TG* triglycerides, *HDL* high-density lipoprotein, *LDL* low-density lipoprotein, *SBP* systolic blood pressure, *DBP* diastolic blood pressure

### Muscle function testing - maximal voluntary contraction

**Fig. 2:**
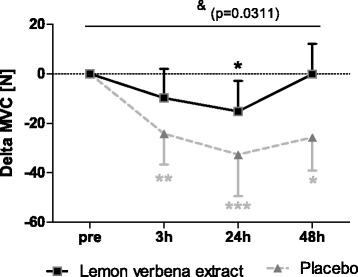
Maximal voluntary contraction. Delta MVC [N] (Mean ± 95% CI; product effect: *p* = 0.0311) * indicating significance against baseline; & indicating significant group differeneces

MVC was significantly less reduced after exercise in the lemon verbena group than in the placebo group (*p* = 0.0311), with significant time effect (*p* = 0.0051), (Fig. 2, Table 3). In the lemon verbena group, muscle strength was completely back to baseline after 48 h, whereas strength was still reduced at that time point in the placebo group.

**Table 3:** Statistical results within (one way-ANOVA) and between groups (Linear mixed models with repeated measures or in case of Ret. pain generalized linear mixed model with poisson distribution for count data) for maximal voluntary contraction (MVC), movement induced pain (VAS), retrospective pain (Ret. pain), creatin kinase (CK), gluthation peroxidase (GPxP) and interleukine-6 (IL-6)

	Within group		Between group	
	Lemon verbena	Placebo	Product effect	Time effect
MVC	0.005	<0.0001	0.0311	0.0051
VAS	<0.0001	<0.0001	0.0788	<0.0001
Ret. pain	<0.0001	<0.0001	0.782	<0.0001
CK	<0.0001	<0.0001	0.9412	<0.0001
GPxP	0.204	0.5895	0.0681	0.0624

### Perceived muscle soreness

**Fig. 3:**
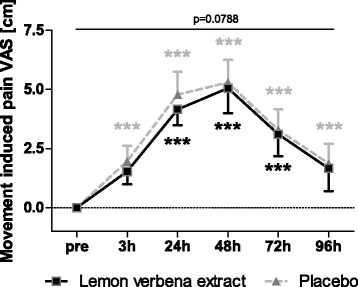
Movement induced pain. Movement induced pain (VAS) [cm] (Mean ± 95% CI; product effect: *p* = 0.0788) * indicating significance against baseline

**Fig. 4:**
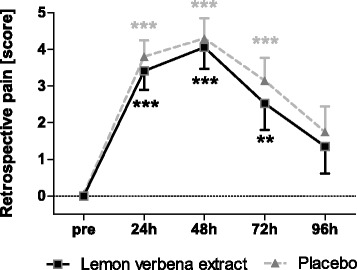
Retrospective pain. Retrospective pain [score] (Mean ± 95% CI; product effect: *p* = 0.7820) * indicating significance against baseline

Movement induced pain (VAS) in the lemon verbena group was less pronounced by trend (*p* = 0.0788) with a significant time effect (*p* < 0.0001) in comparison to the placebo group (Fig. 3, Table 3). Retrospective pain was comparable between groups without significant difference (Fig. 4). Both groups showed significant increase in soreness 24, 48, and 72 h after exercise in relation to pre-exercise.

### Biochemical analyses

#### Creatine kinase

**Fig. 5:**
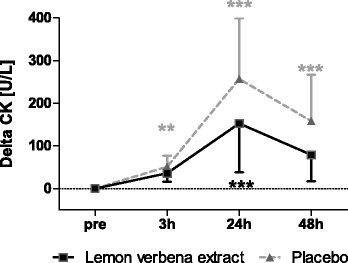
Creatine kinase. Delta Creatine kinase [U/L] (Mean ± 95% CI; product effect: *p* = 0.9412) * indicating significance against baseline

Exercise-induced CK was not significantly different between groups (*p* = 0.9412 with significant time effect (p < 0.0001) (Fig. 5, Table 3).

In the lemon verbena group, CK was significantly increased above baseline at 24 h but not 3- or 48-h post-exercise. In contrast, the placebo group showed significant elevations of CK at all three time points relative to baseline.

#### Glutathione peroxidase

**Fig. 6:**
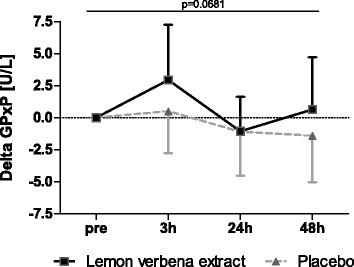
Glutathione peroxidase. Delta Glutathione peroxidase [U/L] (Mean ± 95% CI; product effect: *p* = 0.0681) * indicating significance against baseline

The GPxP activity within lemon verbena group was increased by trend compared to placebo group (*p* = 0.0681), with time effect also by trend (*p* = 0.0624), (Fig. 6, Table 3).

#### Interleukin 6

Only distinct increase of IL-6 could be observed after exercise, without significant differences but high inter-individual variability. Differences between groups were not significant (*p* = 0.5824).

### Safety and tolerability

Blood chemistry, vital signs, adverse events, and concomitant medication did not indicate any safety concerns over 15 days. There was no statistical difference between the two groups for adverse events (*p* = 0.231). The most frequent adverse events were headache (lemon verbena: 5%, placebo: 18%) and common cold (lemon verbena 23%, placebo: 23%). None of the adverse events were serious or related to the study products. The intervention was well tolerated.

## Discussion

The aim of this study was to investigate the effects of supplementing with 400 mg of lemon verbena extract (Recoverben®) on muscle strength and recovery in healthy, moderately active adults. We found that consumption of lemon verbena significantly attenuated loss of muscle strength compared to placebo. Muscle strength loss is considered a reliable and valid functional marker for assessing muscle damage [1]. Therefore, our preliminary findings suggest that lemon verbena may reduce exercise-induced muscle damage.

Muscle strength was reduced by 21% in placebo group, which is within the expected range of 20–50% and recovery was not completed until two days post exercise. It is generally accepted that two to seven days are necessary for full recovery following exercise induced muscle damage [1]. Compared to placebo, lemon verbena extract significantly (*p* = 0.0311) buffered strength loss after exercise. MVC in the lemon verbena group was reduced by 11%, which is defined as mild muscle damage [1]. Furthermore, complete recovery was reached after 48 h. Based on these findings, lemon verbena appears to not only speed recovery, but also reduce fatigue directly after exercise.

These results were reflected by findings for perceived muscle soreness. Movement induced pain, which estimated actual perceived pain showed discrimination between study groups with slight superiority of lemon verbena extract by trend. The less pronounced muscle damage, seen by significantly less reduction of MVC, seems to be reflected by less perceived pain under lemon verbena extract if compared to placebo. Maximum of muscle soreness was reported 48 h after exercise, fitting to the general knowledge that muscle soreness peaks 24 h or 48 h after damaging exercise [1]. The extent of muscle soreness was medium for both groups, supporting that the exercise protocol caused mild to moderate muscle damage. After 96 h, subjects were, on average, not completely painless, even if muscle strength at that time was already recovered in this group. However, the same phenomenon has already been observed by others [9, 33].

Increasing concentration of CK in the blood is an indication of muscle damage, being frequently used in sports nutrition studies [9, 35]. The time course of CK increase peaked at 24 h after exercise, which is comparable to findings reported in literature [9, 35]. Exercise-induced increases in CK are known to exhibit high interindividual variability, with some people showing large increases (responders) and others showing only moderate increases (non-responders) [1]. In our study, high levels of interindividual variation in CK concentrations were present, which could explain why we failed to observe a significant between-group difference despite other markers of muscle damage, such as MVC, favoring the lemon verbena group.

Many research studies have shown that supplementation with dietary polyphenols has the potential to positively influence symptoms of exercise-induced muscle damage [2, 5, 8–10, 41, 19]. However, underlying processes are still unclear and it is not sure if antioxidative effects are the primarily mechanisms [41]. Furthermore, the benefit of reducing oxidative stress has been discussed diversely [41, 42]. Increased of oxidative stress can lead to progressive cell damage and decline in physical function [42, 43]. However, ROS act as biological stimuli in cellular processes of adaption to training [41, 42] and cells can adapt to repetitive increases of ROS by improving antioxidant capacity [44, 45]. During the current study, glutathione peroxidase in plasma was selected as a parameter to supply information about antioxidative capacity. Our results indicate an activation of the antioxidative defense under lemon verbena extract by up-regulating GPxP shortly after exercise. In contrast to this, baseline GPxP was not increased by supplementation with lemon verbena extract. Therefore, it appears, that supplementation with lemon verbena extract strengthens the antioxidative defense system and enables effective counteraction of oxidative stress, but only if needed. Both groups experienced significant exercise-induced increases in IL-6 without significant difference between one another. Some evidence suggests that changes in IL-6 depend in part on exercise intensity and duration [1, 46, 40]. It is possible that the exhaustive exercise protocol used in our study was not intense and/or long enough to elicit meaningful changes in IL-6 that could have been effected by lemon verbena supplementation. Similar results were found in other human studies investigating natural ingredients for effects of muscle strength and muscle damage, such as ashwagandha extract [47], curcumin [48], pomegranate extract [10], and blueberry [2]. These natural ingredients are high in polyphenols, a trait shared by lemon verbena. It has been proposed, that polyphenols could be useful to prevent muscle damage or improve recovery [4]. The major biological functions of polyphenols are as antioxidants and anti-inflammatory agents. Enhanced production of vasodilation factors and the inhibition of synthesis of vasoconstrictors have also been shown [49]. These could be additionally beneficial by improving tissue oxygen supply and removal of metabolic waste products. The proprietary lemon verbena extract (Recoverben®) investigated in the current study has shown anti-inflammatory effects [26] and is characterized by a high polyphenol content and high ORAC level. Therefore, the observed reduction in muscle strength loss and indicated accelerated recovery in the present study might be explained by lemon verbenas’ constituents and their ability to prevent or reduce inflammatory processes or reduce oxidative stress.

## Conclusion

In summary, our study showed that ingestion of a 400 mg/day proprietary lemon verbena extract (Recoverben®) resulted in significantly less muscle strength loss in healthy, moderately active adults. Influences by trend on muscle soreness and antioxidative capacity emphasizes the potential of the product accelerating recovery after exhaustive exercise. Larger studies could provide statistical evidence also for the parameter, which only showed improvements by trend in the study.

## Abbreviations

CK: Creatine kinase
DOMS: Delayed onset of muscle soreness
eiMD: Exercise-induced muscle damage
GPx: Glutathione peroxidase
GPxP: Glutathione peroxidase in Plasma
IL-6: Interleukin 6
MVC: Maximal voluntary contraction
ROS: Reactive oxygen species
RPE: Rating of perceived exertion
VAS: Visual analogue scale
